# Molecular Basis Underlying Changes of Brain Entropy and Couplings in Late-life Depression

**DOI:** 10.2174/011570159X385271251129225844

**Published:** 2026-04-16

**Authors:** Meiling Chen, Hongjiang Zhang, Xiaohui Yu, Jie Zhang, Jingmei Zhong, Kexuan Chen, Rong Zi, Zhe Wu, Zhiyuan Wang, Kunhua Wu, Jiaojian Wang, Heng Shao, Ying Zhao, Baosheng Zhu

**Affiliations:** 1 Department of Clinical Psychology, The First People’s Hospital of Yunnan Province, Kunming, China;; 2 Kunming University of Science and Technology Affiliated Hospital, Kunming, China;; 3 Faculty of Life Science and Technology, Kunming University of Science and Technology, Kunming, China;; 4 Department of MRI, The First People’s Hospital of Yunnan Province, Kunming, China;; 5 Kunming Medical University, Kunming, China;; 6 State Key Laboratory of Primate Biomedical Research, Institute of Primate Translational Medicine, Kunming University of Science and Technology, Kunming, China;; 7 Yunnan Key Laboratory of Primate Biomedical Research, Kunming, China;; 8 Medical School, Kunming University of Science and Technology, Kunming, China;; 9 Department of Medical Genetics, The First People's Hospital of Yunnan Province, Kunming, China;; 10 Department of Medical Genetics, NHC Key Laboratory of Healthy Birth and Birth Defect Prevention in Western China, Kunming, China;; 11 Department of Gynecology, the First People’s Hospital of Yunnan Province, Kunming, China;; 12 Department of Geriatrics, The First People’s Hospital of Yunnan Province, Kunming, China

**Keywords:** Late-life depression, brain entropy, functional connectivity, gene enrichment analysis, neurotransmitter, temporoparietal junction (TPJ)

## Abstract

**Introduction:**

Late-life depression (LLD) with high prevalence accelerates cognitive impairment and becomes a risk factor for dementia, yet the pathogenesis of LLD is still largely unclear. Delineating the neuromolecular mechanism of LLD is of great significance to its etiology, early diagnosis, and precision treatment.

**Methods:**

This study included 35 patients with LLD and 41 age-matched healthy controls (HCs). Brain entropy (BEN) and functional connectivity (FC) were used to assess the abnormalities in brain functional system irregularity and couplings in LLD, using resting-state functional magnetic resonance imaging. Additionally, transcriptome and neurotransmitter data were employed to investigate the neuromolecular mechanisms underlying these changes.

**Results:**

Compared with HCs, patients with LLD exhibited significantly reduced BEN in the temporoparietal junction (TPJ) and decreased FC between TPJ and the middle frontal gyrus (MFG). Moreover, the changes of BEN were closely associated with the genes’ expression profiles, which were involved in endocannabinoid, adrenergic, oxytocin, and cGMP-PKG signaling, glutamatergic, GABAergic, dopaminergic, cholinergic, serotonergic synapses, long-term potentiation, and long-term depression. We also found that the changes of BEN and FC were correlated with neurotransmitter-receptor distribution patterns of serotonin, histamine, acetylcholine, and dopamine.

**Discussion:**

Reduced BEN of TPJ in LLD indicates impaired regional information-processing complexity, potentially driving persistent cognitive deficits and abnormal negative-emotion handling, even in early LLD without overt clinical symptoms. Decreased TPJ-MFG FC disrupts brain network synchronization, weakening MFG-mediated emotion regulation and cognition, and possibly elevating the risk of suicidal ideation. Some genes, such as PRKACA/PRKACB, GNAI1, MAPK-ERK, PLA2G4A, and LXN, along with neurotransmitter receptors, contribute to a more comprehensive understanding of LLD. Altered brain entropy and connectivity in the TPJ-MFG circuit and related neurobiomolecules may underlie the pathogenesis of LLD and provide predictive value for its diagnosis and treatment.

**Conclusion:**

Our study highlights the important role of the TPJ-MFG circuit in the neuropathology of LLD and links the macroscopic functional abnormalities with transcriptome and neurotransmitter to establish the molecular basis. Our findings contribute to understanding the neurobiological basis of LLD and may facilitate future precision therapy.

## 1 INTRODUCTION

According to the report of the World Health Organization (WHO), the number of individuals aged 60 years and older is expected to reach 1.4 billion by 2030 and 2.1 billion by 2050 [1]. As the global population ages, the prevalence of late-life depression (LLD) is increasing [2]. Globally, the average prevalence of LLD is estimated at 35.1% [3], with a prevalence of 20% in China [4]. LLD is strongly linked to increased disability, cognitive decline, elevated suicide rates, and a high risk of dementia [5]. LLD exhibits abnormal brain structure and function due to changes in neurotransmitter systems, brain ischemia, and genetic variations [6-9] suggesting that LLD is a complex brain disorder. Currently, the underlying mechanism of LLD remains poorly understood. Therefore, integrating neuroimaging, transcriptome, and neurotransmitter data is crucial for unraveling the pathophysiological mechanism of LLD.

Neuroimaging studies have demonstrated a reduction in gray matter volume (GMV) and widespread disruption of white matter (WM) integrity in LLD patients [10-12]. With advances in resting-state functional magnetic resonance imaging (rs-fMRI), it is possible to investigate brain regional activity and inter-regional connectivity *in vivo*. Brain entropy (BEN) was developed as a valuable indicator to reflect the brain functional system complexity and irregularity [13]. Higher BEN values indicate more complex, flexible, and dynamic brain systems, while lower BEN values suggest simpler, more rigid systems [14-16]. Abnormal BEN in the prefrontal cortex and hippocampus has been reported in patients with depression [17]. BEN has also been shown to predict the efficacy and prognosis of antidepressant treatments [18]. Another key functional measure of the brain is functional connectivity (FC), which examines the synchronized activity and correlation between different brain regions [19]. FC reflects the transmission and processing of information across brain networks, where different regions interact to form intricate, collaborative systems. Previous studies have identified impairments and disruptions in the functional connectivity of the executive control network (ECN), default mode network (DMN), and salience/emotional processing network (SN) in LLD patients [20-24]. Thus, BEN and FC offer valuable insights into brain functional system irregularity and couplings for assessing the brain's information processing capabilities.

The development of LLD is also influenced by genetic factors [25, 26] which shape brain structure and function. The neurotransmitters, including serotonin, dopamine, and γ-aminobutyric acid (GABA), serve as critical markers for detecting brain chemical metabolic abnormalities in individuals with depression [27]. Advances in constructing full-brain 3D standard atlases for neurotransmitter systems, receptors, and transporters have provided novel tools for exploring the molecular basis underlying brain structural and functional changes [28]. Additionally, the advent of whole-brain gene expression maps, the Allen Human Brain Atlas (AHBA) [29], has opened new avenues for identifying genes with spatial expression patterns linked to neuroimaging phenotypes [30]. Integrating BEN, FC, transcriptome, and neurotransmitter data may pave a new avenue to reveal the neural and molecular basis of LLD.

In this study, we investigated alterations in BEN and FC in LLD patients through rs-fMRI and explored the neuromolecular mechanisms underlying these neuroimaging changes using transcriptome and neurotransmitter-receptor distribution data. We collected rs-fMRI data from 35 LLD patients and 41 healthy controls (HCs) and first analyzed the alterations in BEN and FC in patients with LLD. Next, we performed spatial correlation analysis between statistical maps of BEN, FC, and transcriptomic profiles, neurotransmitter density maps to reveal the molecular basis underlying changes of BEN and FC. Finally, gene enrichment and protein-protein interaction (PPI) analyses were used to uncover the main functions of these genes and hub genes regulating neuroimaging changes.

## 2 MATERIALS AND METHODS

### 2.1 Participants

A total of 76 participants were included in this study: 35 patients with late-life depression and 41 matched HCs. All subjects were recruited from the Clinical Psychology and Geriatrics Departments at the First People's Hospital of Yunnan Province. This study was approved by the local Ethics Committee (KHLL2023-YJ22), and all patients were provided and signed an informed consent form. Inclusion criteria were as follows: participants were aged 60-80 years, right-handed, experiencing a first onset of moderate-major depression according to DSM-5 criteria, had a Hamilton Depression Rating Scale (HAMD-17) score ≥ 17, and had not received any prior treatment for depression. Exclusion criteria included: other psychotic disorders, mental and behavioral disorders due to psychoactive substance use, neurodegenerative diseases, other serious neurological conditions, serious physical illnesses, use of certain medications (*e.g*., non-steroidal anti-inflammatory drugs, sedatives, thyroid hormones, or antipsychotic drugs within the past two months), claustrophobia, and metal implants in the body. LLD patients underwent clinical assessments using the HAMD-17, Hamilton Anxiety Rating Scale (HAMA), Pittsburgh Sleep Quality Index (PSQI), and Montreal Cognitive Assessment (MoCA) scales.

### 2.2 Resting-state fMRI data acquisition and preprocessing

Resting-state fMRI data were collected using a Siemens 3.0T MRI scanner (Magnetom Prisma) with a standard head coil system at the First People's Hospital of Yunnan Province. During the scan, participants remained awake with eyes closed, breathing calmly to stay relaxed. The rs-fMRI scanning parameters were as follows: repetition time (TR) = 1500 ms, echo time (TE) = 30 ms, flip angle = 70º, field of view (FOV) = 192 × 192 mm², matrix size = 64 × 64, voxel size = 3.0 × 3.0 × 3.0 mm³ with 0.75 mm gap, and 250 time points.

The rs-fMRI data preprocessing included the following steps: removing the first 10 volumes, head motion correction, normalization to the Montreal Neurological Institute (MNI) template, smoothing with an 8 mm Gaussian kernel, temporal band-pass filtering (0.01-0.1Hz), and regressing out signals from white matter, global mean, and cerebrospinal fluid. A cubic spline interpolation method was used to correct for data points with head motion exceeding mean framewise displacement (FD) > 0.2 mm to minimize motion artifacts [31-33].

### 2.3 BEN Mapping

BEN mapping, defined using Fuzzy Entropy (FuzzyEn), was performed to quantify the brain system functional irregularity for each subject [13, 34, 35]. FuzzyEn was measured by calculating the negative natural logarithm of the conditional probability that two similar vectors at M consecutive points will remain similar at the next (M + 1) points. A cutoff threshold of 0.2 was applied, with an optimal window length of M = 2 [34, 36]. After obtaining the voxel-wise FuzzyEn map for each subject, a two-sample t-test was conducted to identify differences in BEN between the LLD and HC groups with sex as a covariate, and the significance level was set at *p* < 0.05 using the AlphaSim correction method with voxel-level *p* < 0.001.

### 2.4 FC Analyses

To explore the changes in FC in LLD patients, seed-based functional connectivity analysis was performed. The seed regions were defined based on the brain areas that showed significant differences in BEN in patients with LLD. Differences in FC between LLD patients and healthy controls were statistically analyzed using a two-sample t-test with sex as a covariate. The significance level was set at *p* < 0.05, corrected using a GRF method (cluster-forming threshold at voxel-level *p* > 0.001).

### 2.5 Transcription-neuroimaging Association Analysis

To assess the relationship between BEN, FC changes, and gene expression profiles, we used the Allen Human Brain Atlas (AHBA) dataset, which includes brain samples from six human donors (five males, one female) and 10,027 normalized gene expression data points (http://human.brain-map.org/) [37]. The statistical t-maps of BEN and FC were mapped onto a brain atlas developed by Schaefer *et al.* [38] dividing the brain into 500 subregions. The average t-value of each subregion was computed. The spatial Pearson correlation analysis between each gene’s expression values and the mean t-values of all the 500 regions was conducted. To identify the significantly associated genes with changes of BEN and FC, the significance level was determined using the Bonferroni correction method with *P* < 0.05.

### 2.6 Enrichment Analysis of genes and Protein-protein Interaction Mapping

Gene enrichment analysis was conducted using Metascape (https://metascape.org/) to explore the molecular pathways underlying BEN and FC changes. Gene Ontology (GO) biological processes and Kyoto Encyclopedia of Genes and Genomes (KEGG) pathway enrichment analyses were performed with the following parameters: *P*-value cutoff < 0.01, Min Overlap = 3, and Min Enrichment = 1.5. PPI analysis was performed using STRING v11.0 (https://cn.string-db.org/), with a confidence level of 0.9. Hub genes were identified based on a node degree greater than the mean plus one standard deviation.

### 2.7 Profiling Hub Gene Expression levels in the Human Brain

Neurosynth (https://neurosynth.org/) was used to analyze whole-brain gene expression levels of the top eight hub genes, ranked by node degree associated with BEN and FC changes. Automated anatomical labeling (AAL) [39] was used to define brain subregions, and the average expression levels in each subregion were calculated. The top ten subregions with the highest expression were shown.

### 2.8 Association with Neurotransmitter Density Maps

PET neuroreceptor maps were utilized to assess the spatial Spearman rank correlation between the t-statistic maps of BEN and FC in patients with LLD compared to HC across all brain regions. The neurotransmitter data were accessed from 28 different PET imaging studies of neurotransmitter receptors and transporters in healthy individuals, covering nine neurotransmitter systems: dopamine, norepinephrine, serotonin, acetylcholine, glutamate, GABA, histamine, cannabinoid, and opioid. The group-averaged PET maps of 19 receptors or transporters were shared for research [28] (https://github.com/netneurolab/hansen_receptors/tree/main/data/PET_nifti_images). Statistical significance was determined using a permutation test with 5000 iterations. First, the BEN and FC maps from all LLD and HC subjects were randomly divided into LLD and HC based on their respective group sizes. Next, a two-sample t-test was conducted to evaluate the Spearman rank correlation between the resulting randomized t-maps and the neurotransmitter density maps. The true spatial correlation value was then positioned within the distribution of 5000 randomized spatial correlation values, and the statistical *p*-value was calculated. Bonferroni correction was applied, with a significance threshold set at *p* < 0.05.

## 3 RESULTS

### 3.1 Demographic and Clinical Characteristics

Demographic and clinical features of all participants are summarized in Table **1**. A total of 76 subjects enrolled in the study, comprising 35 LLD patients (9 males, 26 females; mean age = 67.27 ± 7.05 years) and 41 HCs (20 males, 21 females; mean age = 64.27 ± 3.76 years). There was no significant difference in the age between groups (*p* > 0.05), but a significant sex difference was observed (*p <* 0.05), which was taken as a covariate during statistical analysis. Compared to HCs, LLD patients exhibited significantly higher HAMD-17 scores (*p* < 0.05), indicating more severe depressive symptoms. Similarly, HAMA scores were also significantly elevated in the LLD group (*p* < 0.05), suggesting increased levels of anxiety, including both somatic and psychic anxiety. Furthermore, LLD patients had higher PSQI scores (*p* < 0.05) and lower subjective sleep quality (*p* < 0.05), indicating poorer sleep quality compared to HCs. However, there were no significant differences in total MoCA scores or factor scores between the LLD and HC groups (*p* > 0.05).

### 3.2 Differences in BEN between LLD Patients and HCs

To assess differences in BEN between LLD patients and HCs, a two-sample t-test (with sex as a covariate) was performed. BEN was found to be significantly reduced in the TPJ (MNI: [−42, −45, 27]) in LLD patients compared to HCs (Fig. **1**).

### 3.3 Changes in FC between LLD Patients and HCs

Using the TPJ showing a difference in BEN as a seed region, whole-brain functional connectivity analysis was conducted. Significant differences in FC were found between the TPJ and middle frontal gyrus MFG (MNI: [−42, 30, 27], BA45) in LLD patients compared to HCs. Specifically, FC between the two regions was significantly decreased in the LLD group (Fig. **2**).

### 3.4 Molecular Basis Underlying Changes of BEN

To explore the molecular mechanisms underlying BEN changes in LLD, spatial correlation analysis between neuroimaging and transcriptome was performed. A total of 1505 genes were found to be associated with differences in BEN between LLD patients and HCs. KEGG enrichment analysis revealed that these genes were primarily involved in neural pathways related to nerve signaling, synaptic plasticity, and neurotransmitter-receptor interaction. Specific pathways included retrograde endocannabinoid signaling, cGMP-PKG signaling, oxytocin signaling, long-term potentiation, and long-term depression, as well as glutamatergic, GABAergic, dopaminergic, cholinergic, and serotonergic synapses. GO enrichment analysis indicated that these genes were associated with neuronal development, projection, and connectivity, including processes such as dendrite and axon formation, synaptic signal transduction, calcium ion binding, and cellular response to hormonal stimuli. Additionally, a PPI network was constructed based on the genes related to changes in BEN. Eight hub genes with higher node degrees were identified, including PRKACB, GNAI3, GNAI1, MAPK1, GNB1, PRKACG, PRKACA, and MAPK3 (Fig. **3**). Among these, PRKACB showed the highest average expression level in the TPJ, followed by GNAI3, GNAI1, MAPK1, PRKACG, PRKACA, and MAPK3 (Fig. **4A**). The expression of PRKACA, PRKACG, and MAPK1 was relatively high in the limbic system (hippocampus, parahippocampal region, amygdala), while PRKACB, GNAI1, GNAI3, and MAPK1 had higher expression in the basal ganglia (caudate, pallidum, putamen). PRKACG and GNAI3 were highly expressed in the cerebellum, and GNB1, GNAI1, and MAPK3 showed high expression in the frontotemporal lobes (frontal-medial/superior/orbital, temporal-superior/mid/inferior) (Fig. **S1** in Supplementary Materials).

### 3.5 Molecular Basis Underlying Changes in FC

Neuroimaging-transcriptome spatial correlation analysis only identified two genes, PLA2G4A and LXN, associated with changes in FC. PLA2G4A was highly expressed in the frontotemporal lobe (including regions such as the inferior frontal gyrus, temporal pole, and middle frontal gyrus) and the limbic system (insula and Heschl's gyrus). LXN was predominantly expressed in the cerebellum (including vermis regions and cerebellar lobes). Notably, PLA2G4A exhibited higher expression in MFG than LXN (Fig. **4B**).

### 3.6 Neurotransmitter-receptor density Maps Associated with Changes in BEN and FC

To investigate the neurotransmitters associated with changes in BEN and FC, spatial correlation analysis was conducted. Changes in BEN were positively correlated with the density of 5-HT_1B_ (serotonin, r = 0.39), GABA_A/BZ_ (γ-aminobutyric acid, r = 0.36), H_3_ (histamine, r = 0.14), M_1_ (acetylcholine, r = 0.25), and 5-HT_2A_ (serotonin, r = 0.32) receptors (Fig. **5A**). Changes in BEN were also negatively correlated with dopamine transporter (DAT) density (r = -0.38) (Fig. **5A**). Changes in FC were positively correlated with the density of 5-HTT (serotonin transporter, r = 0.14) while negatively correlated with the density of GABA_A/BZ_ and 5-HT_1B_ receptors (r = -0.11 and r = -0.15, respectively) (Fig. **5B**).

## 4 DISCUSSION

Our findings revealed a decrease in BEN in TPJ in LLD patients. BEN characterizes the complexity, randomness, or predictability of dynamic brain processes, symbolizing the brain's capacity to handle complex information [40]. Fuzzy entropy, in particular, offers a more accurate interpretation of entropy. Lower entropy reflects greater predictability and reduced complexity in the brain, whereas higher entropy signifies greater variability and complexity, indicating the enhanced ability of the brain to process information [41]. In healthy older adults, BEN generally increases with age, but this positive correlation reverses as individuals approach the stage of dementia [42]. LLD is frequently observed with neurodegenerative changes in the brain [43]. Unlike major depressive disorder (MDD) in younger individuals, LLD is a distinct condition characterized by both emotional dysfunction and age-related cognitive decline [44]. LLD shows deficits in a series of cognitive domains [45]. Cognitive deficits are a hallmark of LLD and may serve as prodromal symptoms of dementia. These impairments, including deficits in attention, executive function, visuospatial learning, and memory, often persist even after remission of affective symptoms [46, 47]. Studies have shown that these cognitive functions are linked to the activity in the temporal and parietal lobes [48, 49], suggesting that structural and functional abnormalities in the TPJ may contribute to cognitive impairments in LLD, as we observed in this study. In addition, TPJ plays a key role in identifying negative information [50]. Reduced TPJ activity in LLD could impair the ability to process negative emotions, thereby exacerbating depressive symptoms. A common cognitive distortion in individuals with MDD is a pattern of self-blame, where negative events are attributed to oneself and positive events are seen as externally driven [51]. This contrasts with the self-serving bias in healthy individuals, who tend to attribute positive outcomes to themselves and negative outcomes to external factors, thereby protecting their self-esteem and psychological well-being [52]. The negative attribution style is a core feature of depression [53], and the TPJ, which is involved in self-awareness and introspection, may play a role in this distortion. Research suggests that rumination on adverse events may be associated with decreased entropy in the neural processes underlying depression [41]. Patients with LLD often exhibit decreased self-worth, heightened self-criticism, and a lack of hope for the future [54], which may be related to abnormal function of TPJ. Structural and functional abnormalities in TPJ are also associated with neurotransmitter imbalances and inflammatory responses [55], both of which are implicated in depression. Our study found that entropy in the TPJ was reduced in LLD patients compared to HCs, even without cognitive deficits, which may be due to the early stage of LLD, in which clinical symptoms are subtle, but brain functional imaging has already detected potential alterations. Targeting TPJ functional abnormalities might be a promising therapeutic strategy for treating depression in older adults, as early intervention might prevent the onset and progression of LLD.

Our study also found decreased FC between the TPJ and MFG, suggesting a decline in the synchronization and connectivity of the brain circuit to disrupt information transmission. The MFG is critical for emotion regulation and cognitive processing [56]. Reduced TPJ-MFG connectivity likely impairs the MFG's ability to process negative emotions, making LLD patients more vulnerable to these emotions [57]. The MFG is also responsible for cognitive functions, such as attention, working memory, planning, and decision-making. Dysfunction in the MFG may lead to deficits in attention, memory, and decision-making [58], which aligns with the cognitive and emotional symptoms commonly seen in LLD, such as psychomotor retardation, poor insight, and executive dysfunction [59]. Additionally, prior research has linked the cognitive control network (CCN) and the DMN to suicide risk in depressed patients [60]. The TPJ is a core component of the DMN, while the MFG is a crucial part of the CCN. Reduced connectivity between the TPJ and MFG may exacerbate negative mood and cognitive impairments, further increasing the risk of suicidal ideation in LLD patients [5]. In line with this conclusion, a previous study has shown that diminished FC between the MFG and the angular gyrus (part of the TPJ) is associated with severe suicidal ideation in LLD [61]. In summary, abnormalities in the TPJ and TPJ-MFG loop may be closely related to the clinical symptoms of LLD.

In this study, we conducted a neuroimaging-transcriptome spatial correlation analysis to identify genes associated with changes in BEN and FC. These genes were primarily enriched in pathways related to neural signal transduction, neural projection, synaptic plasticity, and neurotransmitter-receptor interaction. These pathways are involved in G protein-coupled signal transduction, intracellular calcium homeostasis, transcriptional regulation, and apoptosis in neural cells. Our findings establish the molecular basis for LLD pathogenesis. For example, the retrograde endocannabinoid signaling pathway has been implicated in both the pathophysiology and genetic factors underlying MDD [62]. The cyclic adenosine monophosphate (cAMP)-dependent protein kinase, also known as protein kinase A (PKA), plays a critical role in intracellular signaling pathways associated with G protein-coupled receptors [63]. The PKA holoenzyme consists of multiple subunit isoforms, including PRKACA and PRKACB. A previous study has demonstrated reduced levels of PRKACA and PRKACB in MDD patients [64]. In animal models of learned helplessness, downregulation of PRKACA and PRKACB in the prefrontal cortex and hippocampus has been observed. These changes may be induced by elevated corticosterone levels, which alter expression levels through the dysregulated hypothalamic-pituitary-adrenal (HPA) axis, a common dysfunction in depression [65]. Zilbermint and colleagues found that activation of PRKACA was relevant to elevated cortisol levels [66]. This is consistent with dysfunction of the HPA axis in depression patients [67]. Defects in serotonin (5-HT) and its receptors are considered major causes of depression, and therefore, genes that regulate this serotonergic-neural pathway play a key role in the development of MDD. Upregulation of GNAI1 has been shown to activate 5-HT1A and 5-HT1B receptors, thereby enhancing serotonergic signaling [68]. The emotional and cognitive symptoms of LLD may also be linked to neurodegenerative changes involving mitogen-activated protein kinase (MAPK) [69]. In our study, MAPK was highly expressed in brain regions associated with depression, including the hippocampus, amygdala, and temporal lobe. The extracellular signal-regulated kinase (ERK), a subfamily of MAPK [70], has been implicated in the pathophysiology of depression. Acute stress increases ERK phosphorylation in the frontal cortex and hippocampus [71], while chronic stress inhibits ERK activity in the hippocampus of a depressed animal model [72]. This inhibition of the ERK pathway limits the translocation of active ERK from the cytoplasm to the nucleus, leading to reduced activity of transcription factors like cyclic AMP response element-binding protein (CREB). The diminished CREB activity, in turn, suppresses the expression of genes associated with depression, contributing to long-term depressive behavior [73].

We also identified that genes of PLA2G4A and LXN were associated with FC changes in TPJ in LLD patients. PLA2G4A was highly expressed in the frontotemporal lobe and limbic system, while LXN was primarily enriched in the cerebellum. PLA2G4A, a major phospholipase in the brain, is upregulated in several neurodegenerative diseases [74, 75]. Early activation of PLA2G4A is likely mediated by upstream kinases such as ERK1/2 and p38 MAPK [76]. LXN is the only known mammalian carboxypeptidase inhibitor, and is initially identified as a marker of neurons in the lateral neocortex of the developing mammalian brain [77]. LXN is also associated with inflammation and can be induced by lipopolysaccharide [78]. Recently, LXN has been found to be a novel modulator for endothelial cell morphology. LXN deficiency significantly ameliorated vascular permeability, vasodilatation, and atherosclerosis in mice [79], indicating that increased LXN is detrimental to vascular homeostasis. Numerous studies have proposed a potential link between endothelial dysfunction (*i.e*., disrupted vascular homeostasis) and depression or suicidal behavior [80, 81]. Some studies also suggested that small vessel disease, systemic microvascular dysfunction (disruption of vascular homeostasis), may contribute to the development of LLD [82]. Overall, our study suggests that LLD-related genes are highly expressed in brain regions such as the frontal lobe, temporal lobe, limbic system, and cerebellum, which are known to be involved in the pathogenesis of LLD [83-85]. These genes and enriched pathways identified in our study may provide valuable biological targets for the precision interventions for LLD.

Our findings also revealed that changes in BEN and FC in LLD patients are associated with neurotransmitter receptor densities. Deficiencies in monoamine neurotransmitters such as 5-HT and dopamine (DA) are widely recognized as major causes of depression. Activation of the 5-HT_2A_ receptor by psychedelic drugs has been shown to promote neural plasticity in the cerebral cortex and alleviate depressive symptoms [86]. Deep brain stimulation (DBS) has also been found to relieve depressive and anxious behavior in rodents, potentially *via* 5-HT_1B_ receptor modulation [87]. Additionally, the M_1_-muscarinic acetylcholine receptor is closely related to cognitive function, particularly memory, and may be linked to LLD. Activation of the M_1_ receptor in the perirhinal cortex enhances synaptic plasticity to improve cognitive function [88]. In MDD patients, GABA in cerebrospinal fluid (CSF) and plasma is relatively lower, and glutamic acid decarboxylase-67 (GAD67) is decreased in the prefrontal cortex of post-mortem brain tissue [89]. The levels of GABA_A_ and GABA_B_ receptors are altered, and transgenic animals with knockout of the GABA receptor exhibit depressive-like behavior. In a word, the neuromolecular mechanism of LLD is very complex and requires more in-depth and systematic research in the future.

## STUDY LIMITATIONS

Our study has several limitations. First, LLD is a complex condition with a high rate of comorbidity. Despite adhering to rigorous inclusion and exclusion criteria, confounding factors may still have influenced our results, suggesting a need for more refined research designs. Second, in our study, we found a significant reduction in BEN in the TPJ and decreased FC of the TPJ-MFG circuit. Given that the transcriptomic and neurotransmitter receptor analyses related to BEN and FC were based on whole-brain spatial distribution patterns, therefore, these molecular data cannot fully account for the localized functional alterations observed in the TPJ or TPJ-MFG circuit and only reflect the overall spatial correlation between average whole-brain BEN and FC changes and the expression profiles of associated genes and neurotransmitter receptors. Additionally, transcriptomic and neurotransmitter data were not obtained directly from our subjects, which may have introduced potential sample differences and biased results. The AHBA transcriptomic data used in this study were derived from brain samples of only six donors, with an imbalanced sex ratio (1 female, 5 males) and predominantly left-brain samples from four donors. These limitations may have affected the results of our spatial correlation analysis. Furthermore, although our study focused on older adults with depression, often accompanied by varying degrees of cognitive impairment, we found no significant differences in MoCA scores between the LLD patients and healthy controls. This may be because cognitive impairment in LLD often manifests as mild cognitive impairment, which might require more sensitive cognitive screening tools. Finally, our current study is a prospective exploration and lacks experimental validation. In the future, we plan to verify the regulatory effects of key genes on BEN and FC through gene knockout/over-expression experiments to further elucidate the causal relationship between these genes and BEN/FC.

## CONCLUSION

In summary, we investigated the neuromolecular mechanisms of LLD by integrating rs-fMRI, transcriptomic data, and neurotransmitter receptor density maps. Patients with LLD exhibited reduced BEN in the TPJ and weakened FC between the TPJ and the MFG. The identified abnormal circuit of TPJ-MFG may serve as an effective target for non-invasive neuromodulation for LLD. The reduced BEN was mainly associated with genes’ expression profiles, including PRKACB, GNAI3, GNAI1, MAPK1, GNB1, PRKACG, PRKACA, and MAPK3, whereas decreased FC was linked to expression profiles of PLA2G4A and LXN. These genes are implicated in synaptic signaling and neurotransmitter pathways, including glutamatergic, GABAergic, dopaminergic, cholinergic, and serotonergic systems, which was further supported by the spatial association analysis between neuroimaging alterations and spatial distribution of receptor densities. The identified neurotransmitters and genes involved in pathways may facilitate future medicine discovery for LLD. Taken together, our findings highlight the important role of the TPJ-MFG circuit in LLD and revealed the molecular basis underlying functional changes of LLD, which will enhance our understanding about the biological underpinnings of LLD and may facilitate early diagnosis and personalized precision therapy.

## Figures and Tables

**Fig. (1):**
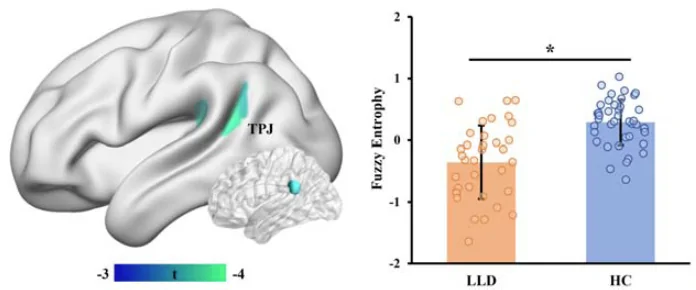
The differences in brain entropy (BEN) of temporoparietal junction (TPJ (MNI: [−42, −45, 27]) in late-life depression (LLD). Patients with LLD showed significantly reduced BEN characterized using fuzzy entropy in TPJ. * represents *p* < 0.05.

**Fig. (2):**
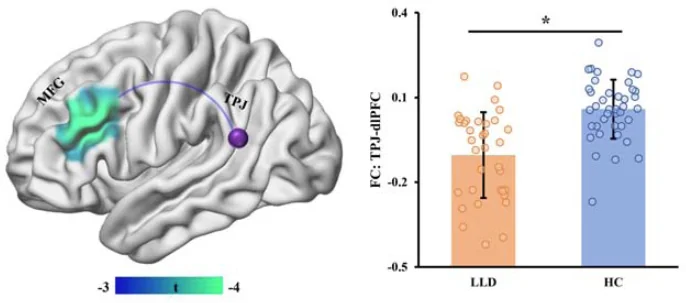
The differences in resting-state functional connectivity (FC) in LLD. Using TPJ as a seed region, a two-sample t-test identified significantly decreased resting-state FC between TPJ and the middle frontal gyrus (MFG (MNI: [−42, 30, 27], BA45) in LLD compared to healthy controls. *represents *p* < 0.05.

**Fig. (3):**
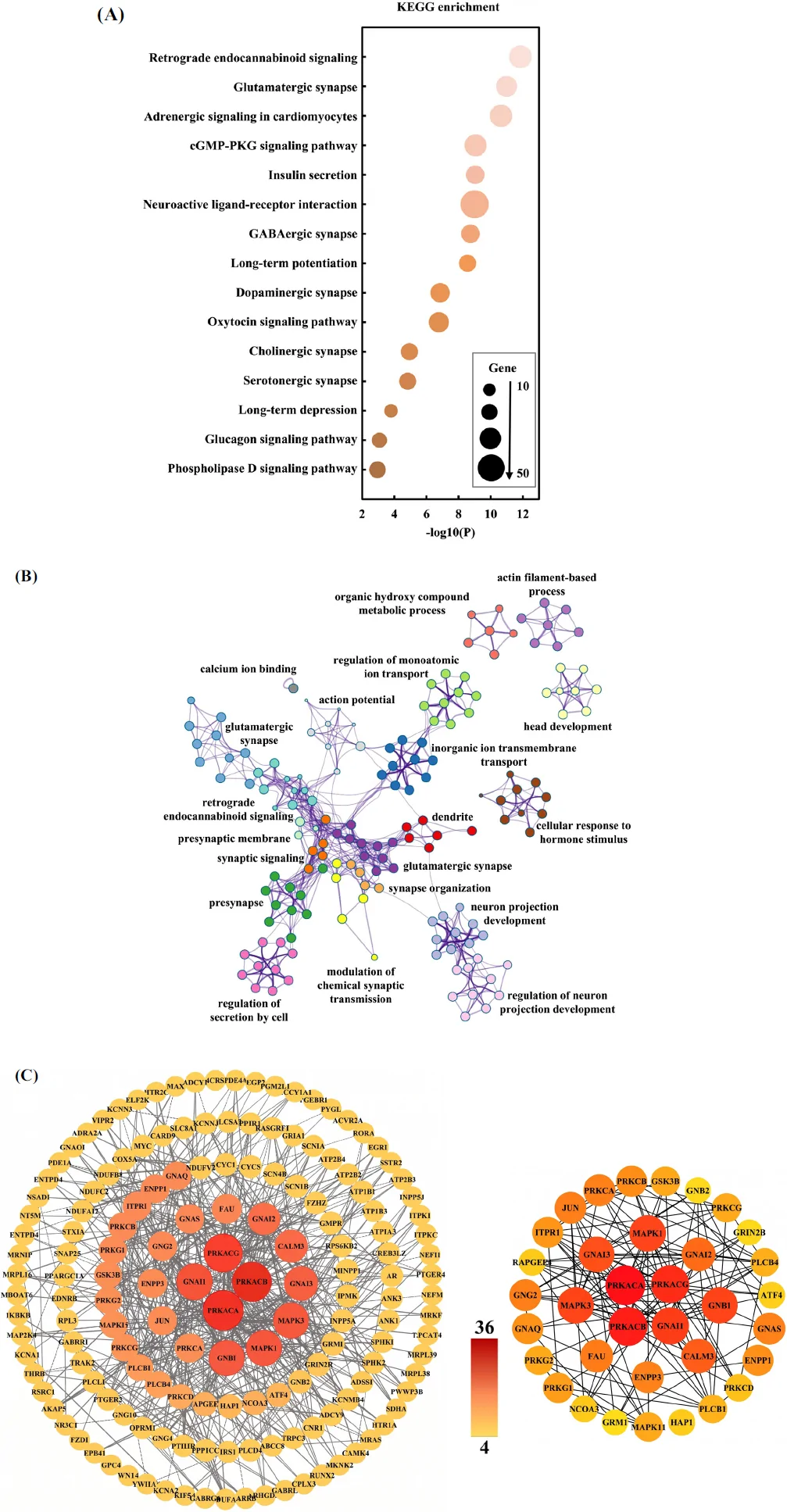
The genes associated with changes in BEN and protein–protein interaction (PPI) analyses. (**A**) The KEGG-enrichment results for genes associated with changes in BEN with *p* < 0.05 (BH-FDR correction). (**B**) The GO-enrichment network of cluster analysis for BEN-related genes is shown. In this graph, if two nodes belong to the same cluster, they are placed close to each other with the same color. (**C**) The upper graph shows the PPI network plot of gene interactions related to changes in BEN. The bottom graph shows the hub genes of the PPI networks. Different colors are used to indicate the size of the degree, with dark red indicating the highest values.

**Fig. (4):**
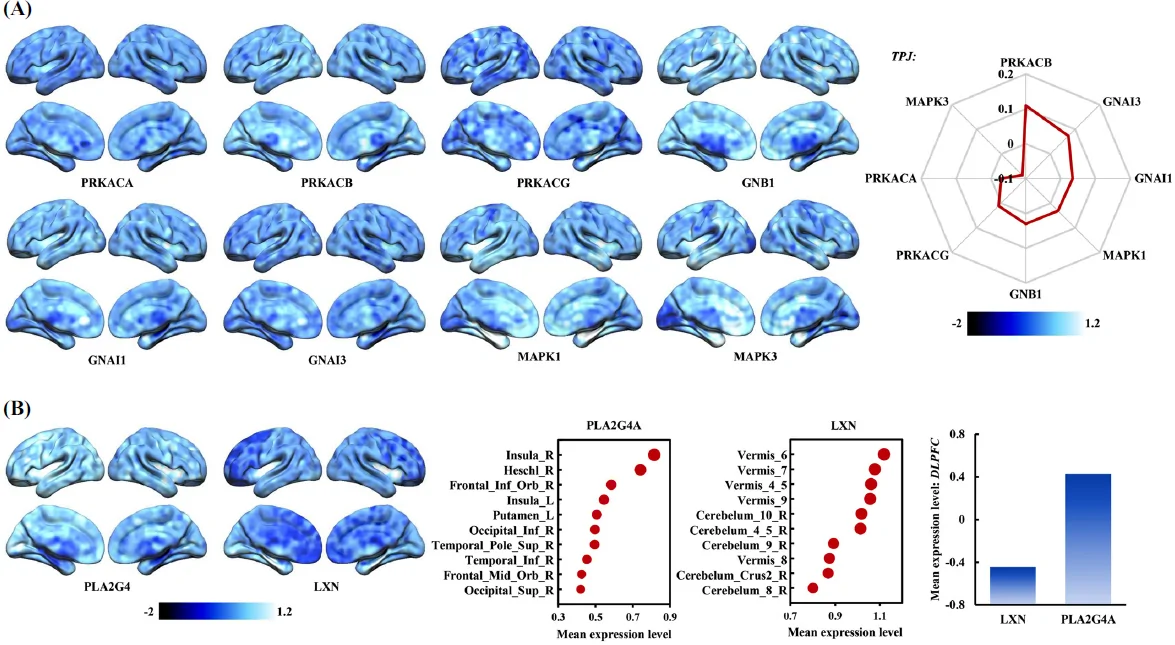
Whole-brain expression level and distribution of the hub genes. (**A**) The whole brain expression level maps of the 8 hub genes associated with the BEN difference were shown (up panel-left section). The mean expression level of these 8 hub genes in TPJ was calculated, and the gene PRKACB showed the highest expression level in this region (up panel-right section). (**B**) The whole brain expression level maps of the 2 genes associated with FC difference were shown (bottom panel-left). In addition, the top 10 brain areas showing high expression level of the two genes were exhibited (bottom panel-middle). The mean expression levels of LXN and PLA2G4A in MFG (bottom panel-right) were calculated, and the gene PLA2G4A was relatively more highly expressed in this region than LXN.

**Fig. (5):**
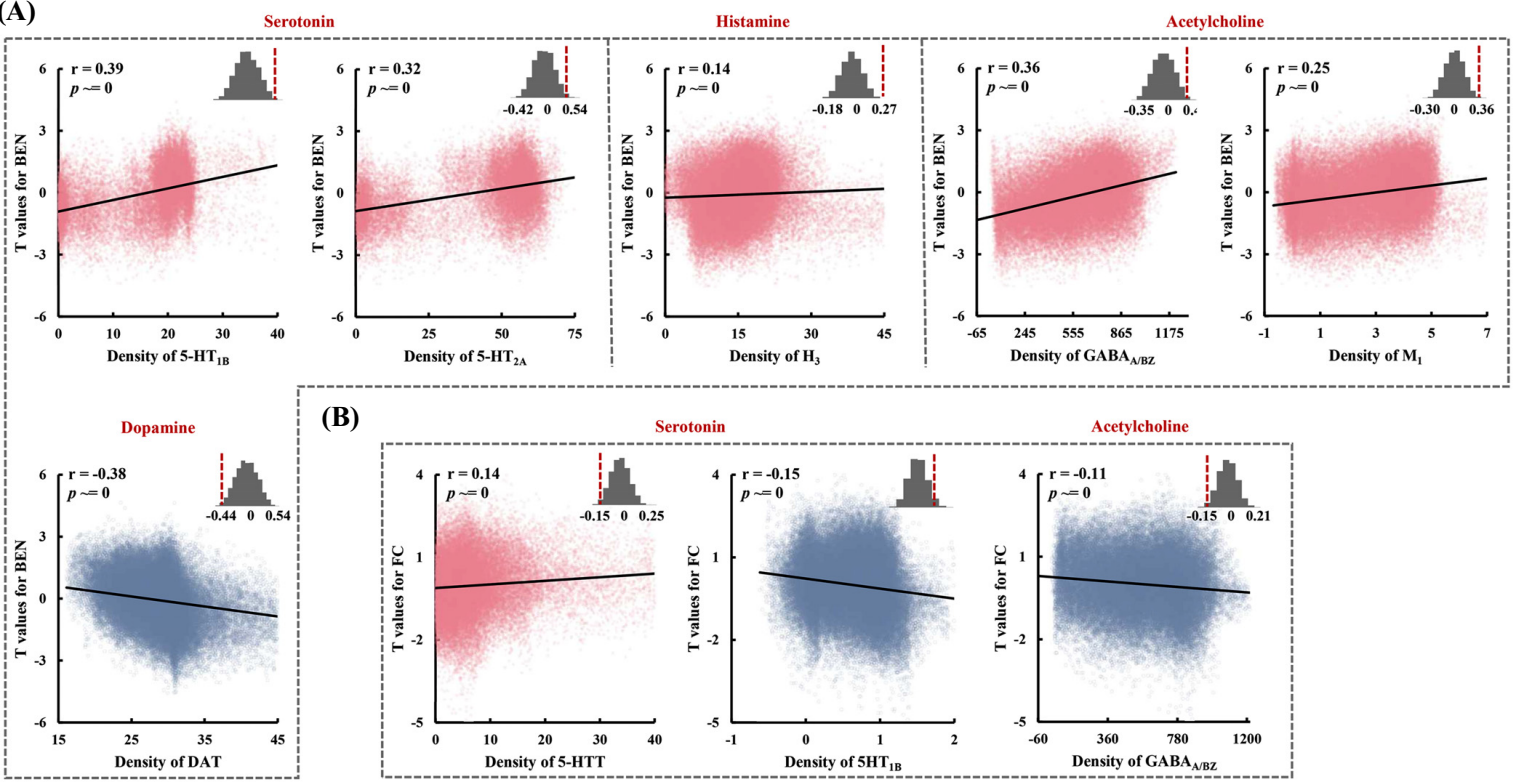
Relationship between neurotransmitter-receptor density and changes in BEN or FC. (**A**) Significantly positive correlations were found between changes in BEN and the densities of 5-HT_1B_, GABA_A/BZ_, H_3_, M_1_, and 5-HT_2A_ (*p* < 0.05), while a significantly negative correlation was found between changes in BEN and the density of DAT (*p* < 0.05). (**B**) A positive correlation was observed between changes in FC and 5-HTT density (*p* < 0.05), while a negative correlation was found between changes in FC and the densities of GABA_A/BZ_ and 5-HT_1B_ (*p* < 0.05).

**Table 1:** Demographic and clinical information for the participants.

**Characteristics**	**LLD** **(35)**	**HC** **(41)**	***P* value**
**Sex (M/F)**	9/26	20/21	0.04
**Age (years)**	**67.27 ± 7.05**	64.27 ± 3.76	0.13
**Hamilton Depression Rating Scale-17 scores**	17.03 ± 5.89	3.88 ± 2.51	9.22×10^-21^
**Hamilton Anxiety Rating Scale**	16.83 ± 6.04	4.59 ± 3.49	2.97×10^-17^
Somatic Anxiety	8.00 ± 3.73	3.02 ± 2.41	9.63×10^-10^
Psychic Anxiety	8.80 ± 3.38	1.56 ± 1.50	1.00×10^-19^
**Pittsburgh Sleep Quality Index**	12.17 ± 4.55	6.51 ± 4.14	2.59×10^-7^
Subjective Sleep Quality	2.03 ± 0.75	1.20 ± 0.87	3.18×10^-5^
Sleep Latency	2.06 ± 0.91	1.34 ± 0.94	1.21×10^-3^
Sleep Duration	2.14 ± 0.97	1.17 ± 0.92	2.77×10^-5^
Habitual Sleep Efficiency	1.74 ± 1.04	0.93 ± 1.10	1.49×10^-3^
Sleep Disturbances	1.86 ± 0.81	1.32 ± 0.72	2.97×10^-3^
Use of Sleeping Medication	1.17 ± 1.32	0.20 ± 0.64	7.29×10^-5^
**MoCA**	23.23 ± 3.73	23.22 ± 3.01	0.99
Visuospatial and executive ability assessment scores	3.71 ± 1.07	3.76 ± 1.11	0.87
Naming Ability Assessment Scores	2.86 ± 0.36	2.98 ± 0.16	0.06
Attention Test Scores	5.00 ± 1.35	4.78 ± 1.11	0.44
Verbal Test Scores	2.20 ± 0.83	2.20 ± 0.90	0.98
Abstraction Test Scores	1.43 ± 0.70	1.20 ± 0.84	0.20
Delayed Recall Test Score	2.29 ± 1.72	2.44 ± 1.30	0.66
Orientation Scores	5.71 ± 0.62	5.73 ± 0.45	0.89

**Note**: Chi-square test was used for sex and two-sample t-test was used for age, HAMD, HAMA, PSQI and MoCA to assess statistical differences. The Kolmogorov-Smirnov-Lilliefors method was used to determine if the quantitative data followed a normal distribution. HAMD: Hamilton Depression Rating Scale, HAMA: Hamilton Anxiety Rating Scale, PSQI: Pittsburgh Sleep Quality Index, MoCA: Montreal Cognitive Assessment, HCs: Healthy controls, LLD: Late-life depression.

## Data Availability

All data generated or analyzed during this study are included in this published article.

## LIST OF ABBREVIATIONS

5-HT: Serotonin (5-hydroxytryptamine)
5-HTT: Serotonin Transporter
AAL: Automated Anatomical Labeling
AHBA: Allen Human Brain Atlas
BEN: Brain Entropy
cAMP: Cyclic Adenosine Monophosphate
CCN: Cognitive Control Network
cGMP-PKG: Cyclic Guanosine Monophosphate - Protein Kinase G Signaling Pathway
CREB: Cyclic AMP Response Element-binding Protein
CSF: Cerebrospinal Fluid
DA: Dopamine
DAT: Dopamine Transporter
DBS: Deep Brain Stimulation
DMN: Default Mode Network
ECN: Executive Control Network
ERK: Extracellular Signal-regulated Kinase
FC: Functional Connectivity
FuzzyEn: Fuzzy Entropy
GABA: Gamma-aminobutyric Acid
GAD67: Glutamate Decarboxylase-67
GMV: Gray Matter Volume
GO: Gene Ontology
H3: Histamine H3 Receptor
HAMA: Hamilton Anxiety Rating Scale
HAMD: Hamilton Depression Rating Scale
HC/HCs: Healthy Control/Healthy Controls
HPA: Hypothalamic-pituitary-adrenal
KEGG: Kyoto Encyclopedia of Genes and Genomes
LLD: Late-life Depression
M1: Muscarinic Acetylcholine Receptor M1
MAPK: Mitogen-activated Protein Kinase
MDD: Major Depressive Disorder
MFG: Middle Frontal Gyrus
MNI: Montreal Neurological Institute
MoCA: Montreal Cognitive Assessment
PET: Positron Emission Tomography
PKA: Protein Kinase A
PPI: Protein-protein Interaction
PSQI: Pittsburgh Sleep Quality Index
rs-fMRI: Resting-state Functional Magnetic Resonance Imaging
SN: Salience/Emotional Processing Network
TPJ: Temporoparietal Junction
WHO: World Health Organization
WM: White Matter

## STANDARDS OF REPORTING

STROBE guidelines and methodology were followed.
