# Supplementary material for: Molecular Basis Underlying Changes of Brain Entropy and Couplings in Late-life Depression
Source: Curr Neuropharmacol. 2026 Apr 16;24(11):1650–64. doi: 10.2174/011570159X385271251129225844 (PMC13647684; doi:10.2174/011570159X385271251129225844)
Supplement: Supplementary file 1 [file CN-24-11-1650_SD1.pdf]

Supplementary Material

Molecular Basis Underlying Changes of Brain Entropy and Couplings in Late-life Depression

Meiling Chen[1,2,3,#], Hongjiang Zhang[2,4,5,#], Xiaohui Yu[6,7,#], Jie Zhang[2,4,#], Jingmei Zhong[1,2], Kexuan Chen[8], Rong Zi[2,11], Zhe Wu[1,2], Zhiyuan Wang[8], Kunhua Wu[2,4], Jiaojian Wang[6,7], Heng Shao[2,12,*], Ying Zhao[2,4,*] and Baosheng Zhu[2,9,10,*]

[1]Department of Clinical Psychology, The First People's Hospital of Yunnan Province, Kunming, China; [2]Kunming University of Science and Technology Affiliated Hospital, Kunming, China; [3]Faculty of Life Science and Technology, Kunming University of Science and Technology, Kunming, China; [4]Department of MRI, the First People's Hospital of Yunnan Province, Kunming, China; [5]Kunming Medical University, Kunming, China; [6]State Key Laboratory of Primate Biomedical Research, Institute of Primate Translational Medicine, Kunming University of Science and Technology, Kunming, China; [7]Yunnan Key Laboratory of Primate Biomedical Research, Kunming, China; [8]Medical School, Kunming University of Science and Technology, Kunming, China; [9]Department of Medical Genetics, the First People's Hospital of Yunnan Province, Kunming, China; [10]Department of Medical Genetics, NHC Key Laboratory of Healthy Birth and Birth Defect Prevention in Western China, Kunming, China; [11]Department of Gynecology, the First People's Hospital of Yunnan Province, Kunming, China; [12]Department of Geriatrics, The First People's Hospital of Yunnan Province, Kunming, China

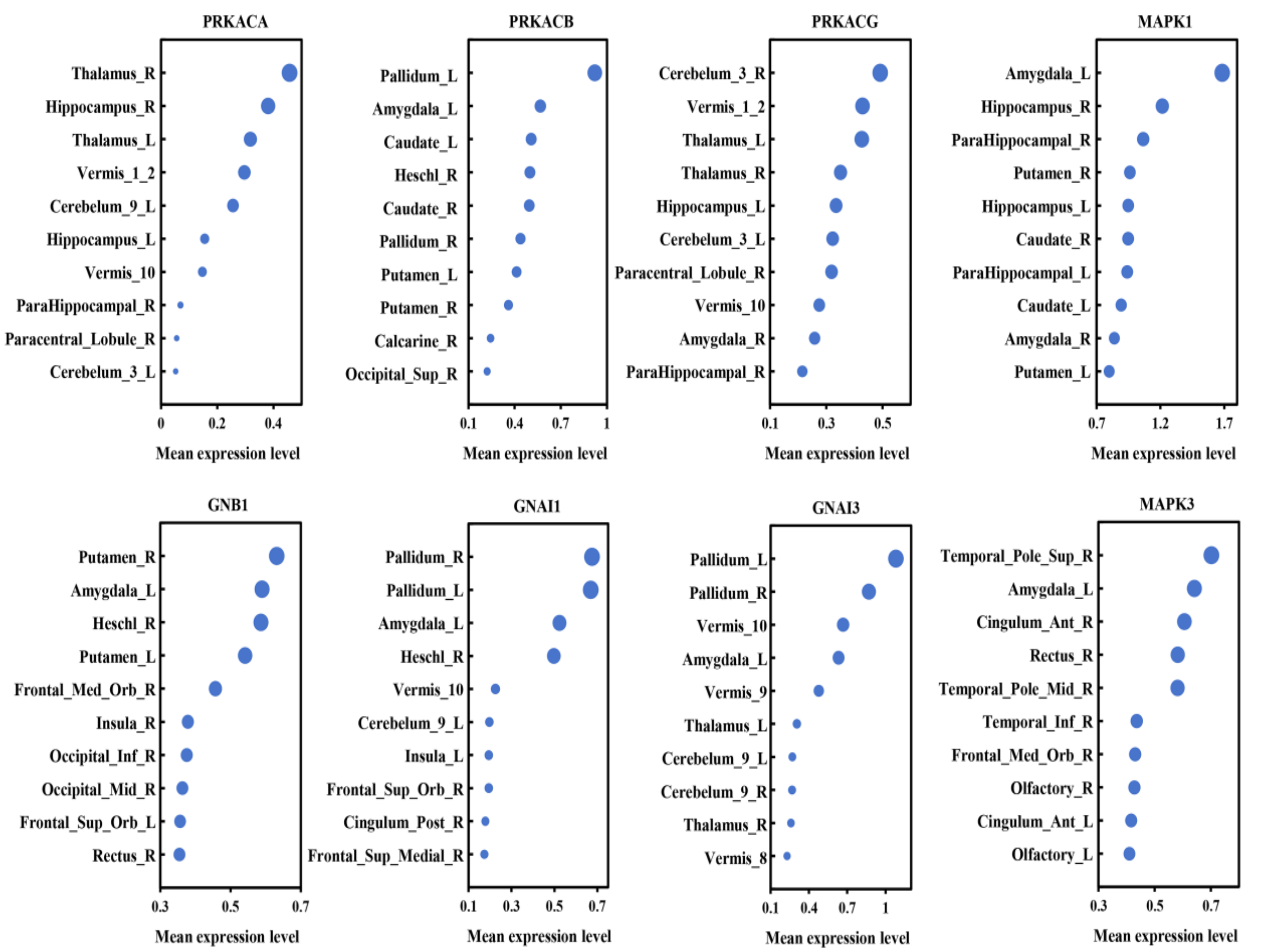

Fig. (S1). The top 10 brain regions exhibiting the highest expression levels of the 8 hub genes associated with changes of BEN were presented. The brain regions were defined using the automated anatomical labeling (AAL) atlas.

1875-6190/26 $65.00+.00 **© 2026 Bentham Science Publishers**
